# Oral Administration of Microencapsulated *B. Longum* BAA-999 and Lycopene Modulates IGF-1/IGF-1R/IGFBP3 Protein Expressions in a Colorectal Murine Model

**DOI:** 10.3390/ijms20174275

**Published:** 2019-08-31

**Authors:** Nancy Valadez-Bustos, Eleazar M. Escamilla-Silva, Francisco J. García-Vázquez, Marco A. Gallegos-Corona, Silvia L. Amaya-Llano, Minerva Ramos-Gómez

**Affiliations:** 1 Graduate Studies in Food Science, School of Chemistry, Autonomous University of Queretaro, Cerro de las Campanas S/N, C.P. 76010 Querétaro, Mexico; 2 Chemical Engineering Department, National Technological of Mexico, Tecnológico y Antonio García Cubas S/N C.P. 38010 Guanajuato, Mexico; 3 Molecular Immunogenetics Laboratory, National Institute of Pediatrics, Insurgentes Sur 3700-C, Cuicuilco, C.P. 04530 Ciudad de México, Mexico; 4 Histoptology Laboratory, School of Medicine, Autonomous University of Queretaro. Clavel 200, Prados de la Capilla, C.P. 76017 Querétaro, Mexico

**Keywords:** *B. longum* microencapsulated, gastrointestinal bacterial distribution, lycopene, IGF-1/IGF-1R/IGFBPs system, colorectal cancer

## Abstract

The Insulin-like growth factor-I/Insulin-like growth factor-I receptor (IGF-1/IGF-1R) system is a major determinant in colorectal cancer (CRC) pathogenesis. Probiotics (*Bifidobacterium longum*, BF) and lycopene (LYC) have been individually researched for their beneficial effects in the prevention of CRC. However, the effect of a combined treatment of microencapsulated BF and LYC on IGF-1/IGF-1R/IGFBPs (Insulin-like growth factor-binding proteins) expression in an azoxymethane (AOM)-dextran sulfate sodium (DSS)-induced CRC model have not been demonstrated. BF was microencapsulated by the spray drying technique, with high viability, and daily gavaged with LYC for 16 weeks to CD-1 mice in an AOM-DSS model. The results indicated that BF- and BF + LYC-treated groups had significantly lower inflammation grade, tumor incidence (13–38%) and adenocarcinoma (13–14%) incidence compared to the AOM + DSS group (80%), whereas LYC treatment only protected against inflammation grade and incidence. Caecal, colonic and fecal pH and β-glucuronidase (β-GA) values were significantly normalized by BF and LYC. Similarly, BF and BF + LYC treatments significantly reduced both the positive rate and expression grade of IGF-1 and IGF-1R proteins and normalized Insulin-like growth factor-binding protein-3 (IGFBP3) expression. Based on intestinal parameters related to the specific colon carcinogenesis in an AOM-DSS-induced model, LYC and microencapsulated BF supplementation resulted in a significant chemopreventive potential through the modulation of IGF-1/IGF-1R system.

## 1. Introduction

Cancer is a genetic disorder characterized by an altered balance between proliferation and mechanisms of cell death. Colorectal cancer (CRC) worldwide ranks third in terms of incidence, but second in terms of mortality. Most CRC risk factors are related to dietary and lifestyle factors, which are increased after high meat consumption due to the stimulation of insulin secretion, and increased fat intake. Carcinogenesis caused by insulin resistance leads to increased cell proliferation and reduced apoptosis [1].

Recently, the evidence highlights the insulin/IGF system as an important molecular target for the pathogenesis, progression and treatment of CRC. Among the important components in CRC, the IGF-1 factor and its receptor (IGF-1R) promote both the growth and malignant transformation of adenomatous polyps. IGF-1 factor is found to be more highly expressed in adenocarcinomas compared to adenomas and normal colon samples from patients, while IGFBP3 is lower in patients than in healthy individuals [2,3]. This strongly suggests a positive and a negative association of these two proteins with CRC risk, respectively. Moreover, IGF-1R is frequently overexpressed in cancer cells, which inhibits apoptosis and enhances cell cycle progression [4]. Overall, the levels of these IGF system components might play a central role in cell-cycle progression, differentiation, and proliferation in CRC. However, the underlying pathophysiological links are still barely understood, and the molecular mechanisms still remain unsolved.

The dietary bioactive compound lycopene (LYC), an acyclic isomer of β-carotene found mainly in tomatoes, exerts excellent antioxidant, singlet oxygen quencher and anti-inflammatory properties, and it has been found to be protective against different types of cancer in animal models and epidemiological interventions, with minimal to no toxic effects along with its pleiotropic effects. LYC has promising chemopreventive effects on CRC by modulating IGF-1 system components through direct and indirect actions on pathways such as the Ras/MAPK and PI3K/AKT/Wnt signaling pathways [2,5,6,7,8]. Increasing evidence from epidemiological studies and in vivo models suggests that probiotics, prebiotics and their combination with bioactive compounds might modulate host resistance against intestinal infections. Prebiotics are non-digestible food carbohydrates that selectively stimulate the growth of probiotics in the colon and potentiate the beneficial effects of these microorganisms providing protective effects against colon cancer development [5]. In this regard, the consumption of probiotics such as *Bifidobacterium longum* (BF) has been suggested.

The anticarcinogenic mechanisms associated with the administration of *B. longum* include: control of cell growth and differentiation, fermentation of non-digestible carbohydrates to produce short-chain fatty acids, modulation of the gut microbiome, reduction of pH caused by the excessive presence of bile acids in feces, inhibition of colon carcinoma cell proliferation as well as induction of apoptosis in colon carcinoma cells [9,10]. A daily intake of at least 1 × 10^7^ viable cells has been suggested as the minimum intake to provide a protective effect. However, several factors have been claimed to affect the viability of probiotics, including heat processing and gastrointestinal conditions [11,12]. Due to this, microencapsulation has been developed to protect the cells in order to ensure their effectiveness and ability to induce beneficial effects in the host [13]. The spray drying encapsulation technique is suitable for heat-sensitive materials, and it gives satisfactory results with minimal loss of viability [14]. Although there are previous studies on the microencapsulation of lactic acid bacteria [15], there is no information from in vivo studies regarding the use of mixtures of anionic polysaccharides as encapsulating agents, used independently without the need to be incorporated into food systems, in addition to the evaluation of the beneficial effects of combined strategies using chemopreventive agents and lactic acid bacteria on carcinogenesis. Therefore, the aim of this work was to evaluate the chemopreventive efficacy of a thermomechanically suitable microencapsulation system for BF using spray drying and its evaluation along the gastrointestinal tract (GIT) co-administrated with LYC, through the expression and modulation of IGF-1/IGF-1R/IGFPs system components and its relationship with intestinal parameters related to the specific colon carcinogenesis azoxymethane (AOM)-dextran sulfate sodium (DSS) model.

## 2. Results

### 2.1. General Observations in the AOM + DSS Carcinogenic Study

In order to evaluate the chemopreventive efficacy of the combined probiotic BF + LYC supplementation in an AOM-DSS model, body weight, and fecal pH values, β-glucuronidase (β-GA) activity and viable BF were determined weekly (Table S1).

There were no significant differences in the initial and final body weights after 16-weeks treatment among the experimental groups (Table 1); although we observed mild differences in weight gains and growth rates, the results indicated that none of the treatments negatively affected animal growth. However, we observed anal discomfort in some mice from week 14. In the AOM + DSS control group, four animals presented rectal bleeding, and five animals developed anal prolapsed, whereas only two animals in the LYC 20 + AOM + DSS group and one animal in each of the BF + LYC 20 + AOM + DSS, and Metformin + AOM + DSS groups developed anal bleeding and prolapse, respectively. In addition, bloody stools were observed from week 15 in the AOM + DSS control group. Animals with these conditions were allocated individually and routinely evaluated by a veterinarian. As for the death in the Metformin + AOM + DSS group (Table 1), this fatality occurred at week 12 and, according to the veterinarian, was not related to the AOM + DSS treatment.

Increased colonic pH and β-GA values have been previously reported in chemical-induced colorectal carcinogenesis models [16]. Similarly, pH and β-GA values were significantly higher in the ceacum, colon and feces from the AOM + DSS control group (Table 1). Interestingly, BF + LYC-treated groups had significantly lower caecal, colonic and fecal pH and β-GA values to those of the AOM + DSS control group, except for caecal pH values. Furthermore, both BF- and LYC-treated groups showed similar caecal, colonic and fecal β-GA and pH values to those of the Normal group, being significantly lower for fecal β-GA than the BF- and BF + LYC 50-treated groups. We also observed that the Metformin-treated group had significantly lower caecal, colonic, and fecal pH and β-GA values than those of the AOM + DSS control, but similar to those values of the Normal group. Overall, the results indicated an improvement by BF and LYC treatments, as well as by metformin administration, in intestinal parameters related to specific colon carcinogenesis in the AOM-DSS model.

### 2.2. Microencapsulated Bifidobacterium Longum Viability and Gastrointestinal Tract (GIT) Distribution During the 16-Week AOM + DSS Carcinogenic Study

**Figure 1:**
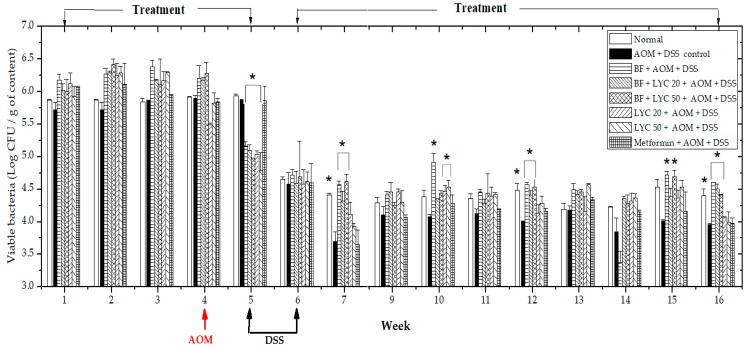
*Bifidobacterium longum* viability in feces of CD-1 mice during the 16 weeks. Values are Log CFU/g of feces. The asterisks (*) indicate significant differences compared to the AOM + DSS control group (Dunnett α = 0.050). Each bar represents the mean ± SEM (*n* = 6–10 animals per group).

In a previous study, the GIT transit of BF BAA-999 (American Type culture collection number-ATCC) microencapsulated by the spray drying technique was monitored for 14 days in an acute model of inflammation in CD-1 mice (2% DSS *v/v* in drinking water, ad libitum). We observed that, the daily intragastrical administration (8.992 × 10^10^ cells·mL^−1^) of a symmetrical sphere of BF (as observed in Scanning Electron Microscopy) with an effective diffusivity had a protective effect on the mucosa, lamina propria, fecal β-GA and body weight in CD-1 mice, while promoting the colonization of BF in luminal content (5.3 to 6.3 Log CFU/g) and its adhesion in the GIT (5.2 to 5.9 Log CFU/g) (data not shown) [17]. As these changes could be related to an anti-inflammatory effect in BF-treated groups, we proceeded to investigate the colonization and protective mechanism of microencapsulated BF in AOM-DSS-treated mice. In the first instance, fecal BF viability was monitored during the 16 weeks (Figure 1); this analysis revealed that fecal levels of the strain in the first four weeks oscillated between 5.7 and 5.9 Log CFU/g for the Normal, AOM + DSS control and Metformin-treated groups; for BF- and BF + LYC-treated groups, the bacteria oscillated between 6.0 and 6.5 Log CFU/g; meanwhile, in LYC-treated groups, the strain viability was between 5.5 and 6.2 Log CFU/g. However, after the administration of AOM and DSS, the levels of BF in all BF and LYC-treated groups were significantly reduced at the 5th week (≈5.1 Log CFU/g) and subsequently retained between 4.3 and 5.0 Log CFU/g from the 7th to the 16th week, which still remained above the limit of detection with significant differences in the Normal, BF, BF + LYC 20 and BF + LYC 50 groups with respect to the AOM + DSS control group.

**Figure 2:**
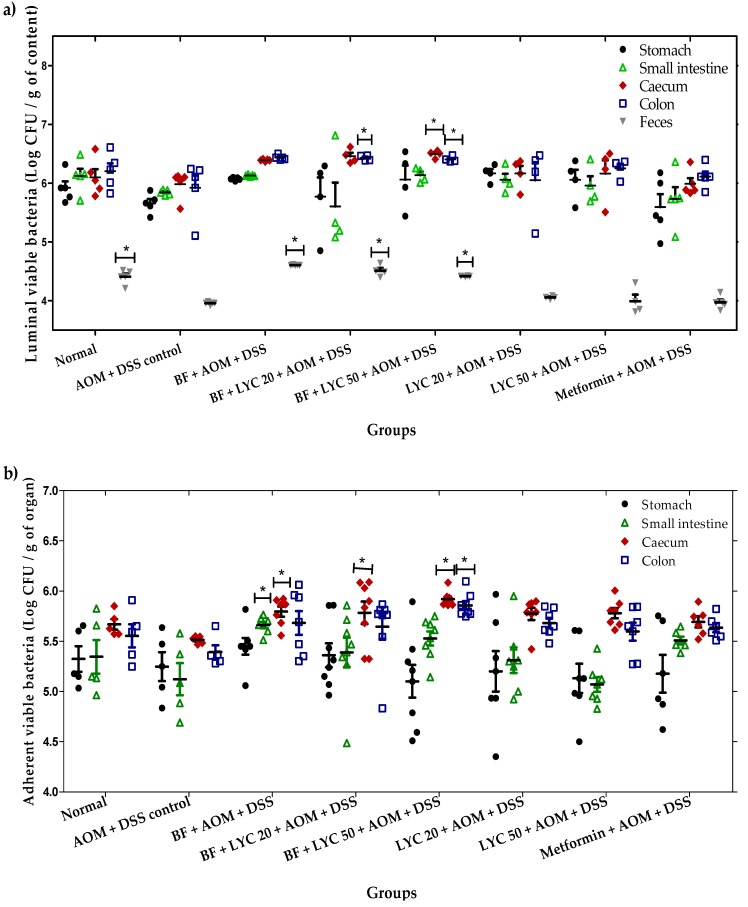
Population of *B. longum* in the stomach, small intestine, caecum, colon, and feces of CD-1 mice at week 16 of the treatment. Values are Log CFU/g of content in the luminal content, feces (**a**) or adherent to the tissue (**b**) at different gastrointestinal sections, as indicated. The asterisks (*) indicate significant differences compared to the AOM + DSS control group (Dunnett α = 0.050). Each bar represents the mean ± SEM (*n* = 6–10 animals per group).

In the second instance, to obtain further information on the preferential colonization site of BF, luminal (Figure 2a) and tissue-adherent (Figure 2b) viable bacteria at different segments of the murine GIT, as well as in feces, were analyzed at week 16. Interestingly, the highest concentrations of the luminal zone parallel to the concentrations in tissue-adherent bacteria were observed in the caecum and colon of animals in the BF, BF + LYC 20, and BF + LYC 50 groups. As a result, the adhesion to intestinal epithelial cells and mucus is a feature that supports the colonization and persistence of BF in the GIT, displaying a protective effect, and as a consequence a lower amount in the feces, as observed in Figure 2a.

### 2.3. Macroscopic Evaluation and Histopathology Classification

Morphological examination of the colonic mucosae of AOM + DSS-treated animals showed the formation of protuberances into the lumen of the colon; flat-type lesions (named early lesions) and pedunculate, sessile, exophytic and endophytic lesions (tumors) were observed in the proximal and distal colon (Table 2). As expected, the Normal group showed the lowest early lesion incidence (10%), and none of the animals developed tumors (*p* < 0.05); meanwhile, the AOM + DSS control group showed 60% and 80% early lesions and tumor incidence, respectively. Although a higher percentage of animals with flat-type lesions were found in BF-, LYC-, and Metformin-treated groups (71–88%), these groups showed lower tumor incidence (by 13–38%, 43–71% and 50%, respectively) and lower mean tumor number, being statistically significant only for BF-treated groups (*p* < 0.05). The metformin group also had a significantly reduced mean tumor number as compared to that of the AOM + DSS control group. The majority of the flat-type lesions and tumors in AOM + DSS-treated groups were found in the distal portion of the colon (67–100%) confirming that this initiation-promotion model predominantly produces tumors in the distal zone [18,19], except for the Metformin + AOM + DSS group, where early lesions and tumors were predominantly found in the proximal colon (85% and 67%, respectively).

**Figure 3:**
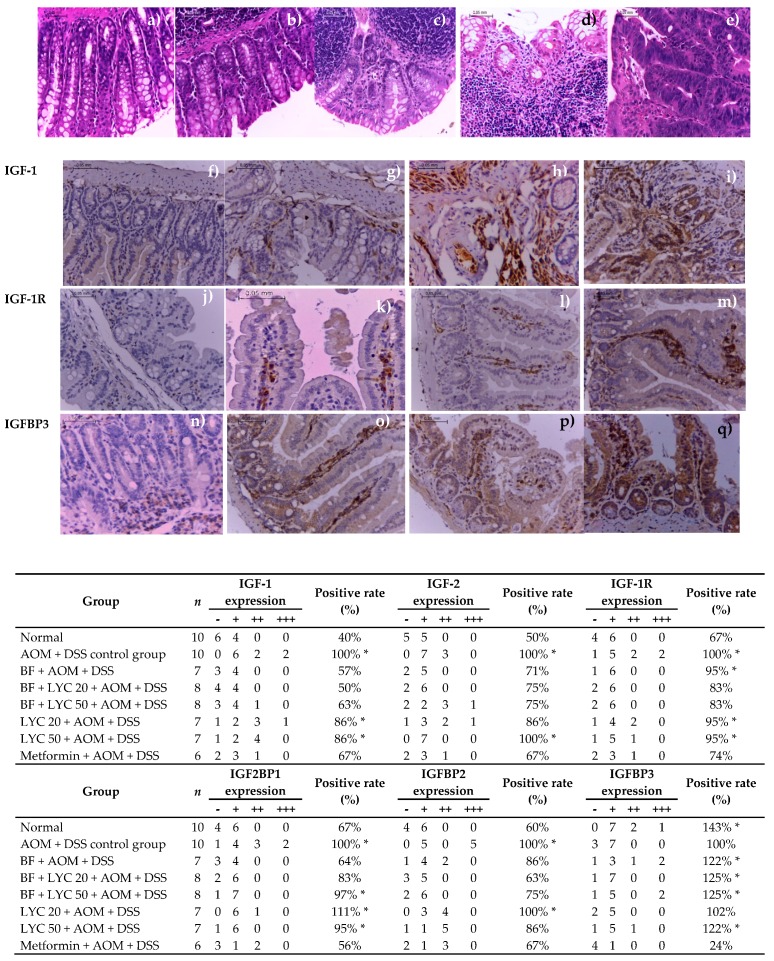
Histological features and IGF-I, IGF-2, IGF-IR, and IGF-binding proteins (IGFBPs) protein expressions in colonic tissues of AOM-DSS-treated mice. H&E staining: (**a**) Normal tissue; (**b**) Low grade inflammation (+); (**c**) Medium grade inflammation (++); (**d**) High grade inflammation (+++); (**e**) Adenocarcinoma. Immunostaining: (**f**,**j**,**n**) Negative protein expressions were <5% (−); (**g**,**k**,**o**) Weakly positive protein expressions were in the range of 5–24% (+); (**h**,**l**,**p**) Positive protein expressions were in the range of 25–50% (++); (**i**,**m**,**q**) Strongly positive protein expressions were >50% (+++). Image captured at ×400). Positive rate (percentage of treated group/AOM + DSS control group). *n*= 6–10 mice per group. * Incidence is statistically significant by chi-square test (*p* < 0.05).

According to the histopathology study, different grades of inflammation and dysplasia were found [19]; however, only inflammation grade and adenocarcinomas were considered (Table 3, Figure 3a–e). As expected, none of animals in the Normal group developed adenocarcinomas and one animal showed inflammation grade ++ (*p* < 0.00002), whereas 80% of the animals in the AOM + DSS group developed adenocarcinomas, and two animals showed inflammation grade +++. BF, BF + LYC- and Metformin-treated groups showed lower adenocarcinoma incidence (by 14%, 13% and 50%, respectively) but higher inflammation incidence (by 75–88%; except for Metformin-treated group). Although BF administration and its combination with LYC showed lower inflammation grades (+ and ++) and significantly protected against adenocarcinomas incidence (*p* < 0.00002), LYC co-administration did not exert synergistic anticarcinogenic protection, but it protected against inflammation (*p* < 0.00002).

Interestingly, colitis was practically absent from AOM + DSS-treated mice after 16 weeks of treatment. However, inflammation was still present at the end of the 16-weeks of experimental period, thus a quantitative and descriptive classification of the damage in the colon, according to lymphocyte infiltration, eosinophils, calceiform cells, epithelial ridges, Peyer′s patches, necrosis, and mitosis presence in tissues of the colon (Table 3) was performed as a strategy to further characterize colonic inflammation in BF- and LYC-treated groups. In addition, BF- and Metformin-treated groups showed similar behaviors, characterized by low lymphocyte infiltration, moderate to intense number of calceiform cells, defined tissue, the presence of epithelial ridges, Peyer′s patches, the absence of necrosis and less that 1% of mitosis, compared to those values of the AOM + DSS control group. Furthermore, BF- and Metformin-treated groups showed similar behaviors to those of Normal group. Conversely, the LYC-groups showed high lymphocyte infiltration, few calceiform cells, the absence of epithelial ridges and Peyer′s patches and the presence of necrosis (Figure 3). Overall the results indicated that the DSS-induced inflammation does not favor the evolution to adenocarcinomas in BF- and Metformin-treated groups.

### 2.4. Modulation of IGF System Components in Colorectal Tissue

Since the IGF-1/IFG-1R system plays a critical role in the development, proliferation, invasion, and survival of colorectal cancer cells [20], we proceeded to demonstrate the synergistic effect of the combined probiotic BF + LYC supplementation on the expression of several IGF system components as an effective strategy for colon cancer chemoprevention. Representative immunostaining expressions of IGF-1, IGF-1R and IGFBP3 proteins are shown in Figure 3f–q, respectively, where a positive expression was observed from brownish yellow to more brownish-yellow granules, and positive rates are expressed in relation to each protein expression of the AOM + DSS control group. As shown in the lower panel of Figure 3, the positive rates of IGF-1, IGF-2 and IGF-1R expression in colonic samples of the Normal group were 60%, 50%, and 33% lower, respectively, as compared to those of colonic samples of the AOM + DSS control group (Chi-square test, *p* < 0.05). Similar to the histopathology analysis, only BF administration and its combination with LYC significantly reduced both the positive rate and expression grade of IGF-1 and IGF-2 proteins; however, LYC co-administration did not further lessen these values. Moreover, BF + LYC-treated groups showed lower IGF-1R expression (Chi-square test, *p* < 0.05). In addition, metformin administration was also significantly effective in reducing IGF-1, IGF-2, and IGF-1R expressions (Figure 3, lower panel).

As IGF-binding proteins (IGFBPs) modulate the amount of bioavailable IGFs in a positive or negative manner [21,22], both the positive rate and expression grade of IGFBPs are shown in the lower panel of Figure 3. In the Normal group, the positive rates of IGF2BP1 and IGFBP2 expression in colonic samples were significantly lower (33 and 40%, respectively), whereas IGFBP3 expression was significantly higher (by 43%, *p* < 0.05) than that of the AOM + DSS control group.

Our results demonstrate that BF and its combination with LYC significantly reduce both the positive rate and expression grade of IGF2BP1 and IGFBP2 proteins (Chi-square test, *p* < 0.05). Moreover, the BF + AOM + DSS group had the lowest IGF2BP1 expression grade, and LYC co-administration had a minor additional effect only on IGFBP2 expression, particularly at the highest concentration (LYC 50). Interestingly, only BF and its combination with LYC significantly increased both the positive rate and expression grade of IGFBP3 protein. On the other hand, metformin administration was effective in reducing the positive rates of IGF2BP1 (by 44%) and IGFBP2 (by 33%) protein expressions (Figure 3, lower panel), but had no effect on normalizing IGFBP3. On the contrary, a drastic decrease in IGFBP3 expression by 76% (Chi-square test, *p* < 0.05) was observed in the Metformin-treated group.

## 3. Discussion

Bifidobacteria are extensively used as probiotics. Alternatively, lycopene (LYC) is a carotenoid that selectively stimulates the growth of probiotics in the GIT and potentiates the beneficial effects of these microorganisms [5]. Using an AOM + DSS model, we investigated a combined probiotic BF + LYC supplementation strategy in the development of CRC by providing thermomechanically enhanced, microencapsulated BF during a 16-week experimental period.

Several factors have been claimed to affect the viability of probiotics, such as gastrointestinal conditions [11,12]. Therefore, microencapsulated BF detectable viability in fecal samples during the experimental period was evaluated by using BSM as selective enumeration of Bifidobacteria. However, the levels of BF in all BF- and LYC-treated groups were significantly reduced at the 5th week after the administration of AOM and DSS probably due to the association between the disturbance of gut ecology and the induction of inflammation by DSS-treatment did not promote colonization of the murine gastrointestinal tract (GIT) by BF from the 5th week. The above may be associated to disruption of the colonic epithelial barrier, ulcerations and heavy infiltration of inflammatory cells into the mucosa with changes in the bile acid enterohepatic circulation and alterations (adaptation or imbalance) in the bacterial flora caused by DSS-treatment [23,24]. Despite its minor colonization of the mouse GIT in the first 4 weeks, BF displayed a protective effect on colorectal cancer when administered daily, maintaining a stable count of BF from the 5th to 16th weeks without statistical difference in each group, according to the analysis by Tukey test at <0.05 (Table S1), confirming that a constant dosage of microencapsulated BF (8.992 × 10^10^ viable cells·mL^−1^) might exert protection in a CRC model. Conversely, Singh and collaborators [25] reported a significant decrease in a *Bifidobacterium* strain of two Log cell counts/g feces in only 24 h after the intragastric administration at day 14 under healthy conditions. 

The adhesion to intestinal epithelial cells and mucus as a feature that supports the colonization and persistence of Bifidobacteria in the GIT; cell surface components that promote colonization and adhesion to the intestinal epithelium include sortase-dependent proteins, exopolysaccharides and lipoproteins, as well as the pili of Bifidobacteria, wich were shown to modulate immune responses. Another aspect contributing to the high or low ability of BF to stably colonize mice might be a better adaptation to its original habitat, i.e., the human infant gut, due to the fact that host adaptation of bifidobacteria has been shown on the level of carbohydrate utilization. BF has the capacity to utilize plant oligo- and polysaccharides that are derived from the diet of the host [24]. As expected, the preferential colonization site of Bifidobacterium strains was in the caecum and colon, with a higher bacterial concentration in the luminal zone [26]. In this regard, our results showed a negative correlation between the β-GA, pH and the bacterial colonization; therefore, the overactivity of the β-GA enzyme and basicity of pH contribute to a lesser bacterial colonization in the luminal zone (*p* < 0.05), while in the tissue-adherent zone, the bacterial colonization was only negatively correlated with the overactivity of β-GA in the caecum, colon, and feces. Our results agree with the anticarcinogenic mechanisms associated to the administration of BF [9,10]. However, this is the first report to demonstrate that LYC- and Metformin-treatments decrease colonic fecal pH and β-GA values in mouse colon carcinogenesis, thus confirming their application as therapeutic intestinal targets in the AOM-DSS model.

In this study, tumor (macroscopic quantitative evaluation) and adenocarcinoma incidence (histopathological classification) induced by AOM + DSS were significantly correlated (*p* < 0.05), thus confirming that these circumscribed masses of cells that project above the surface (polyps) might develop to colorectal carcinomas [23]. Although colitis was practically absent from AOM + DSS-treated mice after 16 weeks of treatment, we still observed higher inflammation grade in colonic samples from all BF- and LYC-treated groups (Table 3). Therefore, we pursued for evaluating whether combined probiotic BF + LYC supplementation strategy could modulate the presence of resident inflammatory cells and others inflammatory scores, and found an improvement in BF- and Metformin-treated groups. This is of major relevance as Tanaka and collaborators [27] have showed that the number of inflammatory cells infiltrated in the colonic neoplasms and lamina propia mucosa, as well as, crypt abscess formation suggest that, these analyses are statistical correlated with the development from premalignancy to malignancy in the colon with inflammation. Similarly, Doulberis and collaborators [28] observed that mice did not show clinical signs of colitis after 3.5 months of experimental period in BALB/cJ mice treated with AOM and DSS (1%, three cycles), but an increased number of inflammatory cells persist in the colon of mice even at 3.5 months after the episodes of colitis induced by DSS. This finding is consistent with our observation that at the end of the 16-weeks of experimental period, mice in BF- and LYC-treated groups had still had resident inflammatory cells. Despite this observation, our results suggest that BF and metformin administration were effective in reducing number of tumors, the composition of resident inflammatory cells and other inflammatory scores, and the incidence of adenocarcinomas in the colon of AOM + DSS treated mice.

The protective role of LYC in the prevention of chronic diseases including cancer has been previously reported [6,7,8], and its co-administration with *B. lactis* + oligofructose/inulin showed a synergistic effect on the initiation phase of 1,2-Dimethylhydrazine (DMH)-induced colon carcinogenesis (total number of Aberrant crypt foci (ACF)) by significantly increasing apoptosis as compared to LYC-, oligofructose + *B. lactis*- and DMH-treated groups [5]. However, it has been previously concluded that dietary LYC is absorbed and then distributed to various tissues, including liver, lung and adipose tissue, thus, deviating its protective role against the development of early and protuberant lesions of the colon [8,29]. This finding is consistent with our observation that at the end of the 16-weeks of experimental period, LYC co-administration did not further improve the protective effect of microencapsulated BF. On the contrary, metformin administration was effective in reducing number of tumors, the composition of resident inflammatory cells and other inflammatory scores, and the incidence of adenocarcinomas in the colon of AOM + DSS treated mice after 16-weeks. Similarly, the chemopreventive effect of metformin has been previously demonstrated [22] and the effects of IGFBP3 knockout and metformin were observed in a murine model of ulcerative colitis, showing significantly reduced colitis [30].

New evidence has demonstrated that the overexpressions of IGF-1 and IGF-1R contribute to the development and progression of colon cancer in patients [4] and in an AOM-DSS-induced colorectal carcinogenesis model [31]. We also found that IGF-1 and IGF-1R expressions correlated with tumor and adenocarcinoma incidence, as well as caecal and fecal pH and β-GA values (*p* < 0.05). However, a negative correlation between IGF-1 and IGF-1R expressions and caecal/colonic-adherent and fecal viable bacteria was observed (*p* < 0.05) (Table S2); thus, the significant protection against adenocarcinoma development observed in BF-treated groups is confirmed. The lower positive rates of IGF-1 in the BF + LYC-treated (up to 50%) and Metformin-treated groups (by 67%) might be of major relevance in our study, since in a previous study, the incidence of tumor growth on the caecum was significantly lower in liver-specific IGF-1-deficient mice orthotopically transplanted with colon 38 adenocarcinoma tissue fragments [32], in which serum IGF-1 levels were 25% of those in control mice.

Because it is currently well established that IGF-1 is mostly bound to IGFBP3, the finding that the positive rate of IGFBP3 was lower in the AOM + DSS control group as compared to the Normal group, while IGF-1 expression was higher in colon samples from the AOM + DSS control group than in animals of the Normal group, confirms the positive and negative correlations (*p* < 0.05) (Table S2), respectively, of these two proteins. Our findings agree with those previously observed in patients with colon cancer [4,33]. Conversely, IGF2BP1 and IGFBP2 expressions significantly correlated with IGF-1 and IGF-2 expressions and adenocarcinoma incidence (*p* < 0.05) (Table S2).

Similar to the higher IGF-2 and IGFBP2 expressions in the AOM + DSS control group, mean IGFBP2 SD scores and expressions were significantly higher in sera and tumors from patients with colonic neoplasia compared to healthy individuals, whereas mean IGF-2 SDs were elevated in Dukes A and Dukes B cases compared with controls, but not in advanced disease [34,35].

As an effective strategy for colon cancer chemoprevention, is worth mentioning that the BF + LYC 20 treatment group showed the best similarity to the Normal group (*p* > 0.05) and a relevant fecal BF viability during the 16 weeks of treatment. Additionally, the preferential colonization site of BF was in the caecum and colon, with a higher bacterial concentration in the luminal zone. Moreover, this group was shown to exert beneficial changes in colonic physiology by decreasing caecal, colonic, and fecal pH, along with β-GA activity, and showing lower inflammation grade, local lymphocyte infiltration, and the suppression of adenocarcinoma incidence compared to those of the AOM + DSS control group. Therefore, it has become clear that the modulation of gut microbiome is likely to be the key element of the health-promoting activity of BF in the colon. Our results, with a low concentration of lycopene, are in agreement with Amir and collaborators [36], who showed a synergistic suppression of HL-60 (promyelocytic leukemia cells) cell growth with a combination of low doses of lycopene and retinoic acid; their results are directly associated with the additive inhibition of cell cycle progression through the G1 phase. Several investigations [37,38] have reported that the absorption of lycopene appears to be more efficient at lower doses due to a complex process involving release from the food matrix, dissolution into mixed chylomicrons, uptake by the liver, distribution to the tissues and secretion into very low-density lipoproteins. In addition, lycopene tends to lose its ability to reduce oxidative damage and exert anticancer effects at higher concentrations. Also, high concentrations of one carotenoid may interfere with the bioavailability of others, leading to imbalance, as occurs between β-carotene and lycopene [39]. Although the mechanism of such a response is unclear, lycopene is presumed to be toxic at higher doses.

**Figure 4:**
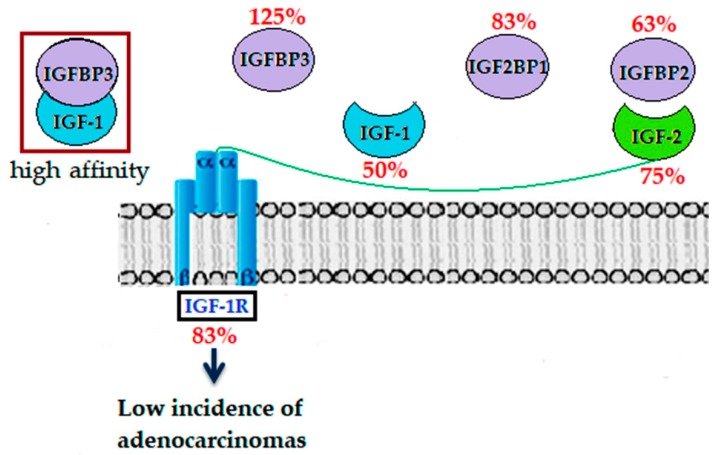
Modulation of the IGF system by BF + LYC 20 in AOM-DSS-treated mice. Restored IGFBP3 expression levels might bind with high affinity to IGF-1, leading to subsequent activation of the signaling pathways by IGF-2 and its binding to IGFBP2, as a result, the low incidence of adenocarcinomas is showed. The percentages in red represent the positive rates expressed in Figure 3.

However, having mentioned that the underlying pathophysiological links with the IGF-1/IGF-1R/IGFPs system are still barely understood and the molecular mechanisms still remain unsolved [4], the BF + LYC 20 supplementation showed strongly decreased expressions of IGF-1, IGFBP2, and IGF-1R; while the IGFBP3 protein expression increased in the colon after AOM + DSS treatment (Figure 4), which has not been previously reported with a new form of as BF microencapsulated using the spray drying technique. In the absence of IGFBP3, the enhanced bioactivity of IGF-1/2 and binding to IGFBP2 might lead to an increase in epithelial proliferation and repair of the mucosal barrier, thereby decreasing DSS-induced inflammation [3]. Due to this, restored IGFBP3 expression levels in BF + LYC-treated groups might lead to binding with high affinity to IGF-1 and then subsequent activation of the signaling pathways by IGF-2 bound to IGFBP2, but at a minor rate. These restored expression levels operating as positive regulators of IGF activity, such as stimulation of the cell cycle and induced apoptosis, thereby inhibiting colon carcinogenesis through the modulation of the PI3K/AKT and MAPK pathways [3,8].

In all studied groups, LYC supplementation did not impede the beneficial actions of microencapsulated BF alone. Although inflammatory cells still persist in the colon of mice even at 16 weeks after the DSS-induced promotion of AOM-initiated carcinogenesis, BF and metformin administration were effective in reducing number of tumors, the composition of resident inflammatory cells, other inflammatory scores, and the incidence of adenocarcinomas in the colon of AOM + DSS treated mice. In our study, we could suggest that the DSS-induced inflammation does not favor the evolution to adenocarcinomas in BF- and Metformin-treated groups and the modulation of the expression of IGF-1/IGF-1R/IGFBP3 protein might be involved in the suppressive mechanisms underlying the evolution to adenocarcinomas in the AOM + DSS model of colon carcinogenesis.

## 4. Materials and Methods

### 4.1. B. Longum (BF) Growth Conditions and its Microencapsulation by the SPRAY Drying Technique

The culture used was the collection of *Bifidobacterium longum* (BF) strain BAA-999, provided by ATCC (American type culture collection, Manassas, VA, USA), wich was freeze-dried; the cellular viability was evaluated in a Neubauer chamber (Marienfeld, Brightline 0.0025 mm^2^, depth 0.100 mm, AUS) with trypan blue as a viability contrast colorant. BF culture was growth with MRS broth (De Man, Rogosa, Sharpe) (Difco, Detroit, MI, USA) supplemented with 0.05% (*w*/*v*) L-cysteine hydrochloride (Sigma, St. Louis, MO, USA) at 37 °C for 24 h, according to Dobrowolski [40]; 2 mL of the activated culture was again inoculated into 100 mL of MRSC broth (MRS supplemented with L-cysteine) at 37 °C for 18 h. The culture was carried out in an anaerobic culture chamber (model 855-ABC, MI, USA) according to Valadez and collaborators [41].

In a previous study, an exhaustive experimental orthogonal design with 72 encapsulated mixtures was designed to selected the mixture with the best physicochemical-dynamical-mechanical-thermal characteristics, based on Young′s modulus, Dynamic Mechanical Analysis, Differential Scanning Calorimetry, diffusivity, and the profile of concentrations through time, as the representative analyses, with high performance under simulated gastrointestinal conditions (data not shown) [41]. In the present study, the cell pellets were re-suspended in the optimal encapsulated material mixture formulated with 1.6% sodium alginate/0.8% gum arabic/2.5% phosphate starch according to Valadez and collaborators [41]. The spray drying technique was performed using a Büchi B-290 spray dryer (Flawil, SWT), according to Lian and collaborators [42] with the following modifications to the operating conditions: temperature of 120 °C for inlet air, 50 °C for outlet air, 95% aspiration, a feed flow rate of 9.6 × 10^−4^ kg·s^−1^ and atomizing air flow of 1.3 × 10^−4^ m^3^·s^−1^ with a supply nozzle diameter of 0.7 mm. The microencapsulated BF were stored in sterile falcon^®^ tubes (Taizhou, Zhejiang, China) at 4 °C. With these conditions, we maintained bacterial survivability 2 logs above the suggested value in the literature, with this acting as an ideal system for delivering probiotic bacteria to the GIT.

### 4.2. Animal Studies

For the carcinogenesis study, 64 male ICR/CD-1 mice (UNAM, Juriquilla, México) at 5–6 weeks of age (28–30 g) were maintained in a temperature and humidity-controlled facility under the conventional 12 h:12 h light/dark cycle. Water and standard diet (Rodent Lab Chow 5001. Nupec, Querétaro, México) were given ad libitum [43]. The experiments on animals were performed in accordance with the Animal Care and Use protocol, approved by the Ethics Committee of the Autonomous University of Quéretaro (Project identification code CBQ17/097-a 18/005, approved 21 February 2018). After one week of acclimatization, animals were randomly assigned to eight experimental groups. Firstly, animals in groups 1–2 (Normal and AOM + DSS Control, *n* = 10) received only sterile NaCl 0.9%; group 3 (BF, *n* = 7) and groups 4–5 (BF + LYC 20 and BF + LYC 50, *n* = 8) received 8.992 × 10^10^ cells·mL^−1^ of microencapsulated BF dissolved in NaCl 0.9% or LYC (Lyc-O-Mato™, Pittsburgh, USA). LYC was extracted from softgels and homogenized with NaCl 0.9% to provide the corresponding concentration; groups 6–7 (LYC 20 and LYC 50, *n* = 7) received 20 and 50 mg kg^−1^ of LYC, respectively. Metformin (1, 1-dimethylbiguanide hydrochloride) has been reported to provide in vivo and in vitro anti-inflammatory effects through inhibition of NF-κB signaling [22,30,44]; thus, a positive control group was included, and animals in group 8 (Metformin, *n* = 7) received 600 mg·kg^−1^ of metformin (Merck company, Naucalpan de Juárez, Edo de México, MEX), dissolved in NaCl 0.9%. The corresponding treatments were prepared daily and gavaged every morning (0.2 mL total volume) to all mice throughout the 16 weeks, with the exceptions of weeks 5 and 6. After 4 weeks, animals in groups 2–8 were treated with a single subcutaneous injection of azoxymethane (AOM, Sigma, St. Louis, MO, USA) 10 mg·kg^−1^, dissolved in NaCl 0.9%; then, at week 5, 2.0% dextran sulfate sodium (DSS, Sigma, St. Louis, MO, USA) was administered ad libitum for 7 days [19]. Body weights were registered, and fecal pellets were collected once a week during the experimental period (16 weeks). Upon sacrifice, by guillotine decapitation, the stomach, small intestine, cecum, proximal colon, and distal colon were immediately excised, and contents were collected separately for each region and stored at −70 °C for pH and β-GA analyses and to evaluate the BF viability.

### 4.3. B. longum Viability in the Stomach, Small Intestine, Caecum, Colon, and Feces of CD-1 Mice

The BF strain was isolated from fecal or tissue samples from experimental animals. For this purpose, fecal pellets and the luminal contents of the stomach, small intestine, caecum, and colon were weighed and homogenized in 1 mL of phosphate buffer solution (PBS, 10%) by vigorous vortexing. For quantification of tissue-adherent BF in the stomach, small intestine, caecum, and colon, each section was triturated using a polypropylene pestle and homogenized in 1 mL of PBS. Log CFUs were determined by plating serial dilutions in PBS on BSM agar (Bifidus Selective Agar). The appropriateness of the BSM medium for *Bifidobacterium* was proved by the successful genus-specific qPCR amplification of tested isolates and the good agreement among plate counting and molecular methods [45]. Besides, previous investigations support the proposed use of BSM as a selective medium for the enumeration of *B. longum* spp. from in vivo models [46]. A single colony was repeatedly restreaked on BSM agar to ensure clonality. The incubation was carried out in an anaerobic culture chamber (model 855-ABC, MI, USA) at 37 °C for 72 h, according to Dobrowolski [40]. The results are expressed as Log CFU/ g of content in the luminal content, feces or adherent to the tissue.

### 4.4. β-Glucuronidase Activity (β-GA) Assay and pH in the Cecal, Colonic and Fecal Content

Specific β-GA and pH values in the caecal, colonic, and fecales content were determined according to Jenab and Thompson [47].

### 4.5. Macroscopic and Histopathological Analyses

At the end of the experimental period of 16 weeks, all animals were sacrificed and subjected to complete autopsy. The stomach, small intestine, colon and caecum were removed, flushed with sterile cold saline solution, and opened longitudinally. The colon was divided into proximal and distal sections and inspected for macroscopic pathological lesions. Macroscopic lesions were fixed in 10% buffered formalin for 6 h and embedded in paraffin blocks; histological procedures for subsequent H&E staining and tumor classification were realized according to Perše and Cerar [48]. Lesions of the colon were classified as normal, inflammation (grade: +, ++, and +++), and adenocarcinomas [23]. All other samples were frozen with liquid nitrogen and stored at −70 °C until analyses.

### 4.6. Protein Expressions of the IGF System by Immunohistochemistry Technique

The colon tissues in paraffin blocks, obtained from the histopathology analysis, were cut into 4-μm slices. Immunostaining expressions of IGF-1 (W18, SC-74116 catalog number), IGF-2 (8H1, SC-293176 catalog number), IGF-1R (2C8, SC-463 catalog number), IGF2BP1 (D-9, SC-166344 catalog number), IGFBP2 (G-4, SC-515134 catalog number), and IGFBP3 (B-5, SC-365936 catalog number) were determined with mouse monoclonal antibodies (Santa Cruz Biotechnology, Inc., CA, USA) according to the procedures reported by Han and collaborators [4], with the following modifications: through deparaffinization at 60 °C, the specimens were submerged in EZ Prep (1X) solution (10%) for 5 min. The samples used for the expression of IGF-1, IGF-1R, IGFBP2, and IGFBP3 were unmasked with sodium citrate 10 mM (pH 6.0); the samples used for the expressions of IGF-2 and IGF2BP1 were unmasked with Tris/EDTA (pH 9.0) in order to optimize unmasking. All the specimens were heated for 5 min at 20 Lb of pressure and 1 atm. Subsequently, the specimens were immersed in 0.9% hydrogen peroxide for 5 min to eliminate activities of endogenous peroxidase for antigen retrieval, rinsed with sterile water and submerged in 10% PBS for 4 min. Then, the specimens were incubated with the corresponding monoclonal antibodies (ratio 1:50) at 37 °C for 45 min, followed by the addition of the biotinylated second antibody (Novocastra Post Primary, Leica, IL, USA) and incubation at 37 °C for 30 min. Subsequently, in order to detect and visualize protein expression levels, specimens were incubated with Novolink Polymer (Leica, IL, USA) at 37 °C for 20 min.

After coloration with diaminobenzidine (DAB) chromogen for 1 min, specimens were rinsed with PBS for 2 min. Hematoxylin was added for counterstaining for 30 s. For the negative control, the primary antibody was replaced with PBS. The classification was according to the proportion of positive cells as follows: single-cell staining and positive cells < 5%, negative (−); small groups of staining and positive cells 5–24%, weakly positive (+); clustered staining and positive cells 25–50%, positive (++); mass staining and positive cells > 50%, strongly positive (+++).

### 4.7. Statistical Analysis

Categorical data are expressed as means ± SEM, using the one-way analysis of variance (ANOVA), followed by the comparison of means by Tukey HSD or Dunnett test. The incidence and histopathological classification of colonic lesions, as well as the positive rates of IGF system expressions, were analyzed by the chi-square test. Correlations were assessed by Pearson correlation coefficient analysis. Statistical analyses were performed using the STATISTICA 7 StatSoft^®^ 2005 (Tulsa, Oklahoma, USA) software, with *p* < 0.05 as a significant difference.

## Figures and Tables

**Table 1:** Final body weight, final body weight gain, pH and β-glucuronidase (β-GA) in the caecum, colon, and feces content at week 16 of the treatment.

Groups	Final Body Weight (g)	Final Body Weight Gain (g)	Caecum		Colon		Feces	
			pH	β-GA ^1^	pH	β-GA ^1^	pH	β-GA ^1^
Normal	39.56 ± 1.01 ^a^	8.41 ± 0.95 ^b^	7.12 ± 0.04 ^b^	3.22 ± 0.33 ^c^	7.21 ± 0.07 ^ab^	2.61 ± 0.16 ^bc^	6.99 ± 0.10 ^b^	5.72 ± 0.03 ^de^
AOM + DSS control	40.46 ± 1.01 ^a^	9.85 ± 0.68 ^b^	7.47 ± 0.04 ^a^	6.56 ± 0.35 ^a^	7.42 ± 0.03 ^a^	5.34 ± 0.53 ^a^	8.08 ± 0.13 ^a^	9.52 ± 0.08 ^a^
BF + AOM + DSS	41.76 ± 1.29 ^a^	12.05 ± 1.03 ^a^	7.20 ± 0.14 ^ab^	3.32 ± 0.34 ^bc^	7.20 ± 0.06 ^ab^	1.85 ± 0.15 ^bc^	6.96 ± 0.10 ^b^	4.88 ± 0.14 ^ef^
BF + LYC 20 + AOM + DSS	41.71 ± 0.97 ^a^	11.71 ± 0.65 ^ab^	7.24 ± 0.04 ^ab^	2.68 ± 0.25 ^c^	7.20 ± 0.06 ^ab^	1.66 ± 0.09 ^c^	7.07 ± 0.20 ^b^	6.71 ± 0.26 ^c^
BF + LYC 50 + AOM + DSS	41.42 ± 0.97 ^a^	11.61 ± 0.71 ^ab^	7.17 ± 0.06 ^ab^	2.37 ± 0.24 ^c^	7.19 ± 0.07 ^b^	2.44 ± 0.27 ^bc^	7.04 ± 0.11 ^b^	4.71 ± 0.12 ^f^
LYC 20 + AOM + DSS	39.78 ± 0.70 ^a^	10.01 ± 0.44 ^ab^	7.39 ± 0.09 ^ab^	3.65 ± 0.24 ^bc^	7.36 ± 0.09 ^ab^	2.73 ± 0.21 ^bc^	7.19 ± 0.13 ^b^	6.26 ± 0.27 ^cd^
LYC 50 + AOM + DSS	41.19 ± 1.29 ^a^	12.10 ± 1.08 ^a^	7.29 ± 0.09 ^ab^	4.74 ± 0.22 ^b^	7.18 ± 0.07 ^b^	3.01 ± 0.31 ^b^	7.18 ± 0.16 ^b^	6.20 ± 0.10 ^cd^
Metformin + AOM + DSS	39.03 ± 1.01 ^a^	9.74 ± 1.14 ^b^	7.27 ± 0.11 ^ab^	2.99 ± 0.31 ^c^	7.28 ± 0.04 ^ab^	2.46 ± 0.27 ^bc^	7.02 ± 0.25 ^b^	7.62 ± 0.33 ^b^

Values are mean ± SEM (*n* = 6–10 animals per group). ^1^ μg phenolphthalein per hour per g of content. Values with different letter(s) within a column are significantly different (Tukey HSD α < 0.05). AOM: azoxymethane; DSS: dextran sulfate sodium; BF: *Bifidobacterium longum*; LYC: lycopene.

**Table 2:** Anticarcinogenic effect of *B. longum* microencapsulates on the macroscopic quantitative classification of colonic lesions induced with AOM and DSS in male CD-1 mice.

Group	*n*	Incidence of Early Lesions ^a^ (%)	Mean Number ^b^	Colon Distribution of Early Lesions ^a^		Incidence of Tumors ^c^ (%)	Mean Number ^b^	Colon Distribution of Tumors ^c^	
				Proximal	Distal			Proximal	Distal
Normal	10	10% *	0.1 ± 0.1 ^b^	1 (100%)	0 (0%)	0% *	0.0 ± 0.0 ^b^	0 (0%)	0 (0%)
AOM + DSS control	10	60%	1.2 ± 0.4 ^ab^	2 (17%)	10 (83%)	80%	4.2 ± 1.0 ^a^	0 (0%)	42 (100%)
BF + AOM + DSS	7	86%	2.4 ± 0.7 ^ab^	1 (6%)	16 (94%)	14% *	0.1 ± 0.1 ^b^	0 (0%)	1 (100%)
BF + LYC 20 AOM + DSS	8	88%	3.0 ± 0.6 ^a^	4 (17%)	20 (83%)	13% *	0.1 ± 0.1 ^b^	0 (0%)	1 (100%)
BF + LYC 50 AOM + DSS	8	75%	2.3 ± 0.7 ^ab^	4 (25)	12 (75%)	38% *	0.4 ± 0.2 ^b^	1 (33%)	2 (67%)
LYC 20 + AOM + DSS	7	71%	1.1 ± 0.5 ^ab^	0 (0%)	8 (100%)	43%	1.7 ± 1.0 ^ab^	0 (0%)	12 (100%)
LYC 50 + AOM + DSS	7	71%	1.3 ± 0.4 ^ab^	3 (33%)	6 (67%)	71%	1.6 ± 0.6 ^ab^	1 (9%)	10 (91%)
Metformin + AOM +DSS	6	83%	2.2 ± 0.9 ^ab^	11 (85%)	2 (15%)	50%	0.5 ± 0.2 ^b^	2 (67%)	1 (33%)

^a^ Macroscopic quantitative evaluation of flat-type lesions. ^b^ Lesion burden/number of mice per group. ^c^ Macroscopic quantitative evaluation of protuberant (pedunculate, sessile, exophytic and endophytic) lesions. Values are mean ± SEM. Values with different letter(s) within a column are significantly different by Tukey test (*p* < 0.05). * Incidence is statistically significant by Chi-square test (*p* < 0.05).

**Table 3:** Histological features and quantitative classification, according to lymphocyte infiltration, eosinophils, calceiform cells, epithelial ridges, Peyer´s patches, necrosis and mitosis, inflammation grade and adenocarcinomas induced with AOM and DSS in male CD-1 mice.

Group	*n*	Normal Tissue	Lymphocyte Infiltration			Eosinophils		Calceiform Cells			Epithelial Ridges		Peyer’s Patches		Defined Tissue	Necrosis	Mitosis	Inflammation Grade			Incidence of Inflammation (%)	Adenocarcinomas
			Low	Medium	High	Yes	No	Few	Moderate	Intense	Yes	No	Yes	No				+	++	+++		
Normal	10	9 (90%) *	None			×				×	×		×		Yes	No	No	0	1	0	10% *	0 (0%) *
AOM + DSS control	10	0 (0%)			×	×		None				×		×	No	Yes	1%	0	0	2	20% *	8 (80%)
BF + AOM + DSS	7	0 (0%)		×		×			×		×		×		Yes	No	−1%	3	3	0	86%	1 (14%) *
BF + LYC 20 AOM + DSS	8	1 (13%)	Focal			×			×		×		×		Yes	Focal	−1%	2	4	0	75%	1 (13%) *
BF + LYC 50 AOM + DSS	8	0 (0%)	×			×			×		×		×		Yes	No	−1%	4	3	0	88%	1 (13%) *
LYC 20 + AOM + DSS	7	0 (0%)			×	×		None				×		×	Focal	Focal	1%	0	2	1	43% *	4 (57%)
LYC 50 + AOM + DSS	7	0 (0%)			×	×		×			×			×	No	Focal	−1%	0	2	1	43% *	4 (57%)
Metformin + AOM + DSS	6	2 (33%)	×			×			×		×		×		Focal	Focal	−1%	1	0	0	17% *	3 (50%) *

Histopathological quantitative classification according to Hematoxylin and Eosin (H&E) staining in colonic tissues (×400). Values are mean (*n* = 6–10 animals per group). * Incidence is statistically significant by Chi-square test (*p* < 0.00002).

## Supplementary Materials

Supplementary materials can be found at https://www.mdpi.com/1422-0067/20/17/4275/s1.

## Abbreviations

ACF	Aberrant crypt foci
AOM	Azoxymethane
BF	Bifidobacterium longum BAA-999
β-GA	β-glucuronidase activity
CRC	Colorectal cancer
DMH	1,2-Dimethylhydrazine
DSS	Sulfate sodium dextran
GIT	Gastrointestinal tract
H&E	Hematoxylin and eosin
IGF-1	Insulin-like growth factor-I
IGF-2	Insulin-like growth factor-II
IGF-1R	Insulin-like growth factor-I receptor
IGF2BP1	Insulin-like growth factor-binding protein-1
IGFBP2	Insulin-like growth factor-binding protein-2
IGFBP3	Insulin-like growth factor-binding protein-3
IGFBPs	Insulin-like growth factor-binding proteins
Log CFU	logarithm of Colony-forming unit
LYC	Lycopene
