# Supplementary material for: Oral Administration of Microencapsulated B. Longum BAA-999 and Lycopene Modulates IGF-1/IGF-1R/IGFBP3 Protein Expressions in a Colorectal Murine Model
Source: Int J Mol Sci. 2019 Aug 31;20(17):4275. doi: 10.3390/ijms20174275 (PMC6747182; doi:10.3390/ijms20174275)
Supplement: Supplementary file 1 [file ijms-20-04275-s001.pdf]

Table 1. Body weight, body weight gain, pH, β-GA and population of *Bifidobacterium longum* during 16 weeks.

Groups	Week	Body weight (g)			Body weight gain (g)			pH			β-GA [1]			Viable BF [2]		
Normal	1	31.15 ± 0.92	e	A	0.00 ± 0.00			7.29 ± 0.30	a	A	5.67 ± 0.07	a	A	5.87 ± 0.02	a	A
	2	32.76 ± 0.72	de	A	1.50 ± 0.17	e	C	7.40 ± 0.08	a	A	5.09 ± 0.18	a	A	5.87 ± 0.02	a	A
	3	32.91 ± 0.84	cde	A	1.70 ± 0.45	e	B	7.23 ± 0.07	a	A	5.26 ± 0.44	a	A	5.85 ± 0.05	a	A
	4	33.60 ± 0.63	bcde	A	2.30 ± 0.30	de	B	7.13 ± 0.03	a	A	5.33 ± 0.30	a	AB	5.91 ± 0.02	a	AB
	5	35.72 ± 1.04	abcd	A	4.60 ± 0.56	cde	B	7.33 ± 0.21	a	A	5.56 ± 0.01	a	CD	5.94 ± 0.03	a	A
	6	36.54 ± 0.82	abcd	A	5.30 ± 0.58	bcd	B	7.23 ± 0.07	a	C	5.12 ± 0.27	a	CD	4.65 ± 0.04	a	A
	7	37.25 ± 0.79	abc	A	6.00 ± 0.73	abc	A	7.28 ± 0.04	a	B	5.55 ± 0.01	a	D	4.42 ± 0.02	a	AB
	9	37.89 ± 0.68	ab	A	6.60 ± 0.86	abc	B	7.24 ± 0.03	a	A	5.33 ± 0.15	a	B	4.29 ± 0.08	ab	A
	10	38.48 ± 0.72	a	AB	7.20 ± 0.76	abc	B	7.21 ± 0.03	a	B	4.87 ± 0.16	a	BC	4.38 ± 0.10	a	BC
	11	39.04 ± 0.81	a	A	7.80 ± 0.77	abc	A	7.34 ± 0.08	a	A	5.02 ± 0.14	a	CD	4.35 ± 0.08	ab	A
	12	37.51 ± 1.07	ab	A	6.40 ± 0.60	abc	B	7.20 ± 0.04	a	A	4.90 ± 0.12	a	CD	4.49 ± 0.10	a	A
	13	39.10 ± 0.92	a	A	7.90 ± 0.71	ab	B	7.10 ± 0.03	a	A	5.71 ± 0.06	a	C	4.19 ± 0.09	b	A
	14	39.72 ± 1.09	a	A	8.60 ± 0.81	a	B	7.32 ± 0.09	a	A	5.70 ± 0.05	a	DE	4.22 ± 0.01	b	A
	15	38.65 ± 1.03	a	A	7.50 ± 0.81	abc	B	7.35 ± 0.13	a	A	5.65 ± 0.04	a	D	4.53 ± 0.12	a	AB
	16	39.56 ± 1.01	a	A	8.40 ± 0.79	ab	B	6.99 ± 0.10	a	DE	5.72 ± 0.03	a	DE	4.41 ± 0.10	a	AB
AOM + DSS control	1	30.62 ± 0.54	e	A	0.00 ± 0.00			7.24 ± 0.25	b	A	5.40 ± 0.26	ef	A	5.72 ± 0.11	a	A
	2	33.55 ± 0.43	de	A	2.90 ± 0.28	e	A	7.20 ± 0.08	b	A	5.09 ± 0.08	f	A	5.72 ± 0.11	a	A
	3	34.63 ± 0.52	cd	A	4.00 ± 0.49	de	A	7.20 ± 0.06	b	A	5.92 ± 0.25	def	A	5.86 ± 0.01	a	A
	4	35.28 ± 0.69	cd	A	4.70 ± 0.47	de	A	7.20 ± 0.10	b	A	5.39 ± 0.02	ef	AB	5.90 ± 0.04	a	AB
	5	36.53 ± 1.00	bcd	A	6.10 ± 0.66	cd	AB	7.74 ± 0.04	ab	A	6.63 ± 0.17	cd	A	5.88 ± 0.00	a	A
	6	37.47 ± 0.70	abc	A	6.90 ± 0.55	bcd	AB	7.85 ± 0.13	a	AB	6.44 ± 0.20	de	A	4.58 ± 0.18	b	A
	7	39.36 ± 0.43	ab	A	8.70 ± 0.62	abc	A	7.98 ± 0.06	a	A	8.27 ± 0.12	b	A	3.70 ± 0.14	c	CD
	9	40.66 ± 0.45	a	A	10.00 ± 0.49	a	A	7.78 ± 0.02	ab	A	8.21 ± 0.25	b	A	4.11 ± 0.12	bc	A

	10	40.83 ± 0.55	a	A	10.20 ± 0.76	a	A	7.88 ± 0.22	a	A	8.24 ± 0.32	b	A	4.08 ± 0.04	bc	C
	11	40.83 ± 0.55	a	A	10.20 ± 0.51	a	A	7.62 ± 0.21	ab	A	7.90 ± 0.21	bc	A	4.13 ± 0.08	bc	A
	12	39.97 ± 0.77	ab	A	9.40 ± 0.75	ab	A	7.54 ± 0.20	ab	A	8.09 ± 0.24	b	A	4.00 ± 0.02	bc	B
	13	40.32 ± 0.60	a	A	9.70 ± 0.50	ab	AB	7.56 ± 0.09	ab	A	8.40 ± 0.24	ab	A	4.18 ± 0.06	bc	A
	14	40.51 ± 0.64	a	A	9.90 ± 0.43	a	AB	7.73 ± 0.06	ab	A	8.33 ± 0.44	ab	A	3.85 ± 0.22	bc	A
	15	40.24 ± 1.41	a	A	10.00 ± 1.07	a	AB	7.55 ± 0.04	ab	A	8.45 ± 0.21	ab	A	4.02 ± 0.02	bc	B
	16	40.46 ± 1.01	a	A	10.00 ± 0.68	a	AB	8.08 ± 0.13	a	A	9.52 ± 0.08	a	A	3.96 ± 0.02	bc	C
BF + AOM + DSS	1	29.71 ± 0.88	e	A	0.00 ± 0.00			7.18 ± 0.30	a	A	5.62 ± 0.04	cd	A	6.18 ± 0.09	ab	A
	2	31.11 ± 0.97	e	A	1.43 ± 0.20	f	C	7.28 ± 0.02	a	A	4.60 ± 0.05	efg	A	6.27 ± 0.08	a	A
	3	32.84 ± 0.95	de	A	3.14 ± 0.40	ef	AB	7.05 ± 0.05	a	A	4.66 ± 0.05	efg	A	6.39 ± 0.10	a	A
	4	33.40 ± 0.97	cde	A	3.71 ± 0.61	def	AB	7.07 ± 0.07	a	A	4.68 ± 0.28	efg	BC	6.21 ± 0.20	a	A
	5	36.39 ± 1.04	bcd	A	6.71 ± 0.81	cde	AB	7.13 ± 0.36	a	A	6.11 ± 0.11	bc	B	5.16 ± 0.07	a	A
	6	37.33 ± 0.78	abcd	A	7.57 ± 0.75	bcd	AB	7.42 ± 0.16	a	ABC	6.65 ± 0.16	ab	A	4.72 ± 0.08	cd	A
	7	37.99 ± 0.96	abc	A	8.29 ± 0.68	abc	A	7.45 ± 0.12	a	AB	6.91 ± 0.05	a	BC	4.57 ± 0.06	cd	AB
	9	38.18 ± 0.81	abc	A	8.43 ± 0.75	abc	AB	7.56 ± 0.19	a	A	4.11 ± 0.22	g	BC	4.42 ± 0.05	cd	A
	10	39.04 ± 0.83	ab	AB	9.29 ± 0.78	abc	AB	7.37 ± 0.07	a	AB	4.62 ± 0.19	efg	BC	4.92 ± 0.14	a	A
	11	39.32 ± 0.84	ab	A	9.57 ± 0.84	abc	A	7.62 ± 0.21	a	A	4.23 ± 0.25	fg	D	4.46 ± 0.05	cd	A
	12	39.91 ± 0.79	ab	A	10.14 ± 0.86	abc	A	7.51 ± 0.22	a	A	4.92 ± 0.24	def	CD	4.58 ± 0.03	cd	A
	13	40.42 ± 1.00	ab	A	10.71 ± 0.99	ab	AB	7.14 ± 0.08	a	A	5.53 ± 0.01	cd	C	4.50 ± 0.09	cd	A
	14	40.52 ± 1.17	ab	A	10.86 ± 0.88	ab	AB	7.51 ± 0.46	a	A	5.36 ± 0.02	cde	EF	3.37 ± 0.18	d	A
	15	41.47 ± 1.29	a	A	11.86 ± 1.14	a	A	7.04 ± 0.24	a	A	4.87 ± 0.05	defg	E	4.72 ± 0.05	bcd	A
	16	41.76 ± 1.29	a	A	12.14 ± 1.03	a	A	6.96 ± 0.10	a	E	4.88 ± 0.14	defg	EF	4.61 ± 0.00	cd	A
BF + LYC 20 + AOM + DSS	1	30.00 ± 0.78	e	A	0.00 ± 0.00			7.31 ± 0.36	abc	A	5.67 ± 0.40	abcd	A	6.02 ± 0.10	a	A
	2	32.40 ± 0.89	de	A	2.25 ± 0.41	f	ABC	7.06 ± 0.11	bc	A	6.01 ± 0.35	abcd	A	6.29 ± 0.03	a	A
	3	33.86 ± 0.82	cde	A	3.75 ± 0.31	ef	A	6.97 ± 0.07	c	A	6.13 ± 0.41	abcd	A	6.18 ± 0.01	a	A
	4	35.05 ± 0.69	bcd	A	4.88 ± 0.48	de	A	7.07 ± 0.04	bc	A	6.06 ± 0.49	abcd	A	6.17 ± 0.04	a	A
	5	37.39 ± 1.00	abc	A	7.38 ± 0.46	cd	A	7.01 ± 0.09	bc	A	5.70 ± 0.06	abcd	BC	5.10 ± 0.08	a	A
	6	38.49 ± 0.67	ab	A	8.38 ± 0.42	bc	A	7.94 ± 0.13	ab	A	6.50 ± 0.11	abcd	A	4.59 ± 0.19	bc	A

	7	38.83 ± 0.96	ab	A	8.88 ± 0.48	bc	A	7.69 ± 0.23	abc	AB	5.08 ± 0.45	cd	D	4.32 ± 0.15	bc	ABC
	9	40.04 ± 0.80	a	A	10.00 ± 0.53	ab	A	7.73 ± 0.28	abc	A	4.80 ± 0.39	d	BC	4.46 ± 0.15	bc	A
	10	40.07 ± 1.15	a	AB	10.13 ± 0.81	ab	AB	7.63 ± 0.22	abc	AB	5.31 ± 0.38	bcd	B	4.35 ± 0.00	bc	BC
	11	40.73 ± 1.31	a	A	10.75 ± 0.84	ab	A	7.53 ± 0.13	abc	A	5.98 ± 0.23	abcd	BC	4.28 ± 0.07	c	A
	12	40.77 ± 0.72	a	A	10.75 ± 0.31	ab	A	8.01 ± 0.10	a	A	6.24 ± 0.37	abcd	B	4.41 ± 0.06	bc	AB
	13	41.60 ± 0.86	a	A	11.50 ± 0.57	a	A	7.44 ± 0.12	abc	A	6.42 ± 0.16	abcd	B	4.42 ± 0.06	bc	A
	14	40.78 ± 0.69	a	A	10.75 ± 0.37	ab	AB	7.11 ± 0.29	abc	A	7.09 ± 0.05	a	BC	4.36 ± 0.04	bc	A
	15	41.90 ± 0.79	a	A	11.88 ± 0.48	a	A	7.03 ± 0.28	bc	A	6.89 ± 0.25	ab	BC	4.39 ± 0.13	bc	AB
	16	41.71 ± 0.97	a	A	11.63 ± 0.65	a	AB	7.07 ± 0.20	bc	C	6.71 ± 0.26	abc	C	4.51 ± 0.07	bc	A
BF + LYC 50 + AOM + DSS	1	29.81 ± 0.48	g	A	0.00 ± 0.00			7.17 ± 0.31	ab	A	5.82 ± 0.30	a	A	6.00 ± 0.19	abc	A
	2	31.50 ± 0.57	fg	A	1.63 ± 0.18	g	BC	7.22 ± 0.27	ab	A	5.67 ± 0.05	ab	A	6.42 ± 0.08	a	A
	3	32.89 ± 0.74	efg	A	3.00 ± 0.33	fg	AB	7.06 ± 0.02	ab	A	5.40 ± 0.20	abc	A	6.12 ± 0.39	ab	A
	4	33.29 ± 0.84	efg	A	3.50 ± 0.27	fg	AB	6.96 ± 0.11	b	A	5.03 ± 0.26	abcde	ABC	6.28 ± 0.17	ab	A
	5	35.40 ± 0.95	def	A	5.75 ± 0.59	ef	AB	7.22 ± 0.45	ab	A	5.18 ± 0.05	abcd	D	4.98 ± 0.00	ab	A
	6	36.21 ± 1.10	cde	A	6.88 ± 0.55	de	AB	7.48 ± 0.12	ab	ABC	5.97 ± 0.20	a	AB	4.70 ± 0.55	cd	A
	7	36.67 ± 0.97	bcde	A	7.25 ± 0.73	cde	A	7.86 ± 0.14	a	AB	5.12 ± 0.18	abcd	D	4.62 ± 0.11	cd	A
	9	38.29 ± 0.86	abcd	A	8.75 ± 0.53	bcd	AB	7.85 ± 0.20	a	A	4.04 ± 0.14	f	C	4.26 ± 0.05	d	A
	10	38.59 ± 0.83	abcd	AB	9.13 ± 0.58	abcd	AB	7.26 ± 0.11	ab	AB	4.04 ± 0.16	f	C	4.44 ± 0.03	d	BC
	11	39.45 ± 0.84	abcd	A	9.75 ± 0.49	abc	A	7.61 ± 0.21	ab	A	4.30 ± 0.31	def	D	4.44 ± 0.30	d	A
	12	39.51 ± 1.09	abcd	A	9.88 ± 0.72	abc	A	7.82 ± 0.19	a	A	4.12 ± 0.33	ef	D	4.53 ± 0.16	d	A
	13	41.54 ± 1.06	a	A	11.75 ± 0.75	a	A	7.45 ± 0.15	ab	A	4.01 ± 0.23	f	D	4.46 ± 0.04	d	A
	14	40.92 ± 1.18	ab	A	11.25 ± 0.80	ab	AB	7.57 ± 0.22	ab	A	4.49 ± 0.09	cdef	F	4.29 ± 0.13	d	A
	15	40.87 ± 0.89	abc	A	11.13 ± 0.61	ab	A	7.23 ± 0.05	ab	A	4.52 ± 0.19	cdef	E	4.69 ± 0.11	abc	A
	16	41.42 ± 0.98	ab	A	11.50 ± 0.71	ab	AB	7.04 ± 0.11	ab	CD	4.71 ± 0.12	bcdef	F	4.42 ± 0.01	d	AB
LYC 20 + AOM + DSS	1	29.77 ± 0.67	e	A	0.00 ± 0.00			7.25 ± 0.24	ab	A	5.74 ± 0.09	bcde	A	6.12 ± 0.17	ab	A
	2	31.54 ± 0.43	de	A	1.71 ± 0.29	f	ABC	7.29 ± 0.00	ab	A	5.78 ± 0.12	bcde	A	6.25 ± 0.18	a	A
	3	33.25 ± 0.47	cd	A	3.43 ± 0.37	ef	AB	7.17 ± 0.07	b	A	5.15 ± 0.20	def	A	6.17 ± 0.14	ab	A
	4	33.40 ± 0.37	cd	A	3.57 ± 0.30	ef	AB	7.20 ± 0.06	ab	A	6.06 ± 0.11	bcd	A	5.49 ± 0.03	c	B

	5	35.64	±	0.64	bc	A	5.86	±	0.46	de	AB	7.27	±	0.22	ab	A	6.00	±	0.11	bcd	BC	5.04	±	0.05	a	A
	6	37.08	±	0.59	ab	A	7.29	±	0.57	bcd	AB	7.51	±	0.17	ab	ABC	5.35	±	0.03	cdef	BC	4.58	±	0.23	bc	A
	7	37.39	±	0.48	ab	A	7.57	±	0.75	abcd	A	7.42	±	0.16	ab	AB	7.22	±	0.51	a	AB	4.12	±	0.18	c	ABCD
	9	37.77	±	0.74	ab	A	8.00	±	0.53	abcd	AB	7.57	±	0.16	ab	A	4.60	±	0.33	ef	BC	4.46	±	0.05	bc	A
	10	36.92	±	0.69	ab	B	7.14	±	0.51	cd	AB	7.43	±	0.09	ab	AB	4.20	±	0.28	f	C	4.46	±	0.09	bc	ABC
	11	38.10	±	0.51	ab	A	8.29	±	0.36	abcd	A	7.93	±	0.09	ab	A	4.16	±	0.25	f	D	4.40	±	0.14	bc	A
	12	38.97	±	0.44	a	A	9.14	±	0.40	abc	AB	8.00	±	0.01	a	A	6.17	±	0.17	bcd	B	4.14	±	0.13	c	AB
	13	39.32	±	0.87	a	A	9.57	±	0.75	abc	AB	7.40	±	0.22	ab	A	6.41	±	0.07	abc	B	4.16	±	0.22	c	A
	14	39.10	±	0.51	a	A	9.29	±	0.42	abc	AB	7.27	±	0.38	ab	A	6.48	±	0.22	ab	CD	4.33	±	0.11	bc	A
	15	39.50	±	0.65	a	A	9.71	±	0.61	ab	AB	7.10	±	0.23	b	A	6.40	±	0.15	abc	C	4.36	±	0.13	bc	AB
	16	39.78	±	0.70	a	A	10.00	±	0.44	a	AB	7.19	±	0.13	ab	B	6.26	±	0.27	abcd	CD	4.06	±	0.02	c	BC
LYC 50 +AOM + DSS	1	29.09	±	0.99	e	A	0.00	±	0.00			7.18	±	0.21	ab	A	5.94	±	0.31	abc	A	5.93	±	0.16	a	A
	2	31.88	±	1.18	de	A	2.86	±	0.46	e	AB	7.40	±	0.10	ab	A	4.35	±	0.09	de	A	6.29	±	0.10	a	A
	3	33.35	±	1.11	cde	A	4.29	±	0.57	de	A	7.23	±	0.09	ab	A	4.29	±	0.09	e	A	6.30	±	0.01	a	A
	4	33.86	±	0.90	bcde	A	4.71	±	0.42	de	A	7.21	±	0.09	ab	A	4.10	±	0.12	e	C	5.83	±	0.16	a	AB
	5	36.80	±	1.13	abcd	A	7.71	±	0.57	cd	A	7.40	±	0.29	ab	A	4.40	±	0.14	de	E	4.79	±	0.27	a	A
	6	37.63	±	1.22	abcd	A	8.57	±	0.78	abcd	A	7.35	±	0.09	ab	ABC	5.16	±	0.17	cd	CD	4.62	±	0.15	bc	A
	7	37.40	±	1.04	abcd	A	8.29	±	0.68	abcd	A	7.72	±	0.06	ab	AB	5.92	±	0.06	abc	CD	3.93	±	0.04	c	BCD
	9	38.68	±	1.10	abc	A	9.57	±	0.65	abc	A	7.52	±	0.15	ab	A	5.29	±	0.13	c	B	4.30	±	0.17	bc	A
	10	38.80	±	1.18	abc	AB	9.71	±	0.87	abc	AB	7.50	±	0.12	ab	AB	5.39	±	0.12	bc	B	4.54	±	0.10	a	AB
	11	39.69	±	1.48	a	A	10.71	±	0.97	abc	A	7.45	±	0.13	ab	A	6.41	±	0.31	a	B	4.42	±	0.03	bc	A
	12	39.62	±	1.28	a	A	10.57	±	1.00	abc	A	7.95	±	0.30	a	A	6.54	±	0.22	a	B	4.28	±	0.12	bc	AB
	13	39.62	±	0.78	ab	A	10.43	±	1.60	abc	AB	7.63	±	0.11	ab	A	6.62	±	0.09	a	B	4.58	±	0.02	a	A
	14	40.74	±	1.36	a	A	11.71	±	1.04	abc	A	7.36	±	0.22	ab	A	6.58	±	0.15	a	CD	4.37	±	0.06	bc	A
	15	41.77	±	1.27	a	A	12.71	±	0.89	a	A	7.01	±	0.26	b	A	6.45	±	0.22	a	C	4.53	±	0.10	a	AB
	16	41.19	±	1.29	a	A	12.14	±	1.08	ab	A	7.18	±	0.16	ab	B	6.20	±	0.10	ab	CD	3.99	±	0.16	c	C
Metformin + AOM +	1	29.29	±	0.81	f	A	0.00	±	0.00			7.12	±	0.45	b	A	6.10	±	0.23	b	A	6.07	±	0.02	a	A
	2	31.05	±	0.67	ef	A	1.71	±	0.29	e	ABC	7.19	±	0.29	ab	A	5.83	±	0.12	bc	A	6.11	±	0.33	a	A

DSS	3	32.67 ± 0.99	def	A	3.43 ± 0.30	de	AB	7.08 ± 0.04	b	A	5.26 ± 0.09	bcde	A	5.95 ± 0.01	a	A
	4	33.15 ± 0.87	cdef	A	3.86 ± 0.34	de	AB	7.11 ± 0.04	b	A	5.69 ± 0.17	bcd	AB	5.84 ± 0.06	a	AB
	5	35.17 ± 0.81	bcde	A	5.86 ± 0.34	cd	AB	7.09 ± 0.20	b	A	5.22 ± 0.04	bcde	D	5.86 ± 0.23	a	A
	6	36.27 ± 0.97	abcde	A	7.00 ± 0.85	bc	AB	7.32 ± 0.02	ab	BC	4.48 ± 0.15	e	D	4.60 ± 0.29	a	A
	7	36.33 ± 1.23	abcd	A	7.14 ± 0.77	bc	A	7.60 ± 0.07	ab	AB	4.69 ± 0.25	de	D	3.65 ± 0.22	b	D
	9	37.78 ± 0.67	abc	A	8.43 ± 0.57	abc	AB	7.45 ± 0.06	ab	A	4.85 ± 0.40	cde	BC	4.07 ± 0.04	ab	A
	10	37.58 ± 0.92	abcd	AB	8.29 ± 0.78	abc	AB	7.79 ± 0.05	a	AB	4.78 ± 0.05	de	BC	4.28 ± 0.14	ab	BC
	11	37.55 ± 0.99	abc	A	8.29 ± 0.84	abc	A	7.42 ± 0.07	ab	A	5.25 ± 0.28	bcde	CD	4.19 ± 0.00	ab	A
	12	38.89 ± 0.89	ab	A	9.67 ± 0.99	ab	AB	7.79 ± 0.02	a	A	5.83 ± 0.17	bc	BC	4.16 ± 0.04	ab	AB
	13	39.03 ± 1.01	ab	A	9.83 ± 0.40	ab	AB	7.48 ± 0.09	ab	A	5.97 ± 0.12	bc	BC	4.34 ± 0.04	ab	A
	14	38.69 ± 1.05	ab	A	9.50 ± 0.67	ab	AB	7.11 ± 0.08	b	A	7.82 ± 0.22	a	AB	4.15 ± 0.03	ab	A
	15	39.65 ± 1.17	a	A	10.50 ± 0.62	a	AB	7.08 ± 0.25	b	A	7.64 ± 0.05	a	B	4.16 ± 0.30	a	AB
	16	39.03 ± 1.01	ab	A	9.83 ± 0.95	ab	AB	7.02 ± 0.25	b	CDE	7.62 ± 0.33	a	B	3.97 ± 0.09	b	C

The analysis is in feces of mice fed *Bifidobacterium longum* microencapsulate. Values are mean ± SEM (n = 6–10 animals per group). [1] μg phenolphthalein per hour per g feces. [2] Log CFU per g feces. Differences of means comparing the sixteen weeks inside each experimental group are expressed in lowercase letters. Each day is compared with the same day of the experimental group respective and differences of measure are expressed in capital letters. Values with different letter(s) within a column are significantly different (Tukey $\alpha < 0.05$).

Table 2. Correlation between IGF-I, IGF-2, IGF-IR, IGFBPs protein expressions and intestinal parameters in AOM-DSS-treated mice.

Variable/ Variable	IGF-1	IGF-2	IGF-1R	IGF2BP1	IGFBP2	IGFPB3
Histopathological classification	0.48	0.15	0.35	0.08	0.19	−0.25
Incidence of tumors	0.35	0.27	0.31	0.32	0.27	−0.22
Incidence of early lessions	−0.02	−0.14	−0.04	0.03	−0.22	−0.07
IGF-1	1.00	0.44	0.63	0.42	0.38	−0.20
IGF-2	0.44	1.00	0.30	0.43	0.33	0.09
IGF-1R	0.63	0.30	1.00	0.41	0.33	−0.22
IGF2BP1	0.42	0.43	0.41	1.00	0.47	0.00
IGFBP2	0.38	0.33	0.33	0.47	1.00	−0.05
IGFPB3	−0.20	0.09	−0.22	0.00	−0.05	1.00
pH caecum	0.37	0.15	0.41	0.27	0.23	−0.07
pH colon	0.24	0.15	0.19	0.39	0.18	−0.01
pH feces	0.36	0.18	0.42	0.33	0.20	−0.23
β-GA caecum	0.40	0.12	0.36	0.27	0.46	−0.24
β-GA colon	0.38	0.13	0.34	0.40	0.43	−0.16
β-GA feces	0.34	0.08	0.36	0.38	0.35	−0.36
Log UFC caecum (Luminal)	−0.32	0.08	−0.26	−0.21	−0.19	0.16
Log UFC colon (Luminal)	−0.12	0.01	0.00	−0.03	−0.29	0.14
Log UFC caecum (Adherent)	−0.32	−0.08	−0.24	−0.23	−0.26	0.09
Log UFC colon (Adherent)	−0.25	0.00	−0.20	−0.18	−0.23	0.04
Feces	−0.42	−0.22	−0.26	−0.33	−0.45	0.42

Correlation of Pearson. Red marked correlations are significant at $p < 0.0500$. Variable = 20. $n = 63$ (6–10 animals per group).
